# Endothelial and cardiac dysfunction in long COVID With cardiovascular symptoms is associated with imbalance in the ADMA–DDAH–NOx pathway

**DOI:** 10.3389/fcvm.2026.1802359

**Published:** 2026-04-21

**Authors:** Mohamed Saad Rakab, Imaduddin Mirza, Mohamed M. Ali, Ammar Khan, Dawood Darbar, Abeer M. Mahmoud

**Affiliations:** 1 Faculty of Medicine, Mansoura University, Mansoura, Egypt; 2 Department of Medicine, Division of Endocrinology, Diabetes, and Metabolism, College of Medicine, University of Illinois Chicago, Chicago, IL, United States; 3 Department of Biobehavioral Nursing Science, College of Nursing, University of Illinois Chicago, Chicago, IL, United States; 4 Department of Medicine, Penn State University, Pennsylvania, PA, United States; 5 Department of Kinesiology and Nutrition, College of Applied Health Sciences, University of Illinois Chicago, Chicago, IL, United States

**Keywords:** cardiac dysfucntion, COVID-19, endothel dysfunction, nitric oxid, vascular dysfucntion

## Abstract

**Background:**

Post-acute sequelae of COVID-19 (PASC) commonly feature lingering symptoms of persistent cardiovascular pathology, yet the mechanisms remain incompletely defined. The ADMA–DDAH–NO*x* axis is a central regulator of endothelial function: ADMA inhibits endothelial NOx synthase, while DDAH clears most circulating ADMA. Although ADMA is linked to acute COVID-19 severity, its regulation in PASC remains largely unknown.

**Methods:**

We performed integrated vascular and cardiac phenotyping in 49 RECOVER participants: never-infected controls (*n* = 10), recovered COVID-19 without persistent symptoms (PASC−, *n* = 20), and PASC with persistent cardiovascular-related symptoms lasting ≥12 weeks post-infection (PASC+, *n* = 19). We measured ADMA, DDAH, NO, inflammatory/coagulation markers, endothelial function [brachial and microvascular flow-mediated dilation (FMD)], and cardiac structure and function using comprehensive echocardiography with speckle-tracking strain.

**Results:**

PASC+ exhibited the highest inflammatory and thrombotic markers, with D-dimer being > 3-fold higher than controls, and hs-CRP nearly threefold higher. PASC+ demonstrated lower NOx and substantially higher ADMA than the other two groups, accompanied by only modest DDAH upregulation, suggesting insufficient counter-regulation. Endothelial function was significantly impaired in the PASC+ group compared to the control and PASC- groups, as evidenced by lower brachial and microvascular FMD. PASC+ individuals exhibited worse longitudinal mechanics and higher levels of hs-troponin and NT-proBNP. Ejection fraction was lower in PASC+ compared with Controls and PASC−.

**Conclusions:**

These findings identify an imbalance in the ADMA–DDAH–NO*x* axis that is associated with endothelial dysfunction and cardiac involvement in cardiovascular-symptom PASC, supporting a potentially targetable pathway for risk stratification and therapeutic investigation.

## Graphical Abstract

**Figure:**
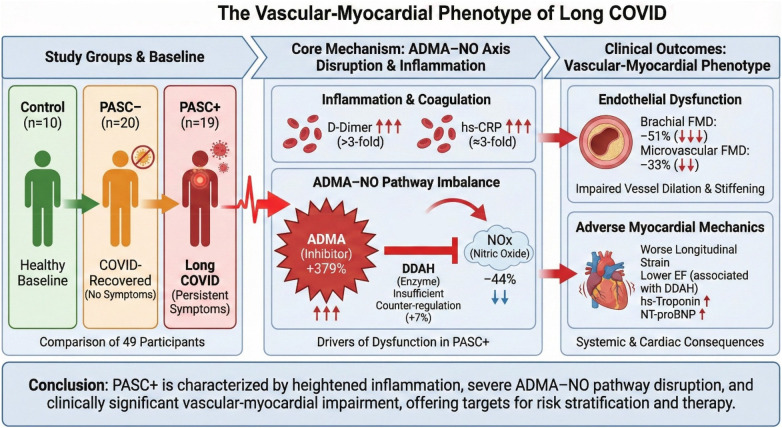

## Introduction

Post-acute sequelae of SARS-CoV-2 infection (PASC, “long COVID”) is a major public health challenge, affecting an estimated 10%–20% of infected individuals and manifesting with fatigue, dyspnea, chest pain, palpitations, dysautonomia, and exercise intolerance months after acute illness (1). Increasing evidence supports that these symptoms frequently reflect persistent multisystem pathology rather than purely functional limitation, with the vasculature and heart among the most consistently implicated organs (2). Defining mechanisms that link SARS-CoV-2 to chronic vascular and myocardial injury is therefore critical for risk stratification and targeted intervention in PASC.

Endothelial dysfunction is a recognized feature of acute COVID-19 and can persist after recovery. Reduced brachial artery flow-mediated dilation (FMD), a non-invasive measure of systemic endothelial function, has been reported during acute infection and in convalescent phases, with impairment correlating with severity and symptom burden in multiple cohorts (3, 4). Parallel biomarker studies describe a persistent endotheliopathy in long COVID, characterized by elevated markers of endothelial activation and a prothrombotic milieu, suggesting ongoing vascular injury beyond viral clearance (5).

At the molecular level, asymmetric dimethylarginine (ADMA) and its metabolizing enzyme dimethylarginine dimethylaminohydrolase (DDAH) are key determinants of nitric oxide (NO) bioavailability and endothelial health (6). ADMA is an endogenous competitive inhibitor of endothelial NO synthase (eNOS); elevated ADMA reduces NO bioavailability, promotes oxidative stress, and contributes to atherothrombotic vascular disease (7). DDAH (DDAH1 and DDAH2) degrades the majority of circulating ADMA, and impaired DDAH function leads to ADMA accumulation and endothelial dysfunction in experimental systems and cardiometabolic disorders (8). While ADMA has been linked to acute COVID-19 severity and outcomes (9), regulation of the ADMA–DDAH–NO*x* axis in PASC is largely unexplored, and DDAH has not been specifically quantified in long COVID despite its central role as the main enzymatic brake on ADMA (6). This pathway is particularly relevant to PASC because, unlike in acute COVID-19 where elevated ADMA may largely reflect the intensity of systemic illness, persistent dysregulation of ADMA clearance and nitric oxide bioavailability after viral resolution could directly contribute to prolonged endothelial dysfunction, microvascular impairment, and chronic cardiovascular symptoms (9). Thus, the ADMA–DDAH–NO*x* axis represents not only a marker of prior acute injury, but also a mechanistic link to long-term vascular sequelae.

To address this gap, we conducted an integrated vascular and cardiac phenotyping study in RECOVER participants comparing symptomatic PASC (PASC+), recovered COVID-19 without persistent symptoms (PASC−), and never-infected Controls. We hypothesized that PASC+participants would exhibit a characteristic imbalance in the ADMA–DDAH–NOx pathway associated with worse endothelial function and evidence of cardiac involvement.

## Methods

### Study design and participant recruitment

We conducted a single-center cross-sectional substudy of participants enrolled in the NIH RECOVER adult observational cohort at the University of Illinois Chicago (UIC) between June 2022 and April 2023, including SARS-CoV-2–infected and uninfected individuals (10). Infected participants met WHO criteria for suspected, probable, or confirmed COVID-19 (11). The index date for infected participants was the date of first positive test or symptom onset; for uninfected participants, the index date corresponded to a documented negative SARS-CoV-2 test. The sample size was based on feasibility and the number of eligible participants available at the UIC site during the study period who completed the study assessments. This observational study was reported in accordance with the Strengthening the Reporting of Observational Studies in Epidemiology (STROBE) guidelines; the completed checklist is provided as Supplementary Table S1.

Participants were classified as PASC+ if they reported persistent or newly developed cardiovascular-related symptoms lasting ≥12 weeks after acute infection, including chest pain/tightness, palpitations, exertional fatigue/exercise intolerance, tachycardia or inappropriate sinus tachycardia, orthostatic intolerance/POTS-like symptoms, dyspnea on exertion not explained by pulmonary pathology, or peripheral edema suggestive of cardiovascular involvement. PASC− participants had prior infection but reported full recovery without ongoing symptoms. Never-infected controls were classified according to the RECOVER protocol and had no known history of SARS-CoV-2 infection, no prior positive diagnostic testing, no prior COVID-19-compatible illness history, and a documented negative SARS-CoV-2 test at the index date. Participants with known pre-existing cardiovascular disease, including prior myocardial infarction, heart failure, clinically significant arrhythmia, cardiomyopathy, or other documented cardiac disorders, were excluded, with preference for individuals without known major pre-existing cardiometabolic or systemic illnesses expected to substantially affect vascular or cardiac phenotyping. Baseline medical history was obtained at screening from participant report and available medical record review to confirm absence of known cardiovascular pathology before SARS-CoV-2 infection.

The time from index date to the study visit ranged from 6 to 12 months, with 92% of participants assessed between 6 and 9 months after the index infection. All participants were recruited from the Greater Chicago metropolitan area. Race/ethnicity were self-reported using standardized categories. The study was approved by the University of Illinois Chicago IRB (protocol #2022-0899, secondary to RECOVER IRB #2021-1287) and conducted in accordance with the Declaration of Helsinki. All participants provided written informed consent.

### Clinical characteristics and routine laboratories

Anthropometric measures (weight, BMI, waist/hip circumferences, waist-to-hip ratio) and vital signs were recorded. Fasting glucose and HbA1c were quantified using standard protocols, and lipid profiles were measured using enzymatic assays as detailed in the original draft Methods (12–14). Routine blood counts, vitamin D, and liver/kidney function tests were obtained via clinical laboratory testing and electronic health records.

### Biomarker assays

NOx was determined using a nitrate/nitrite assay. hs-CRP, D-dimer, ADMA, DDAH, TGF-*β*, hs-troponin-I, NT-proBNP, ACTH, and cortisol were measured by commercially available assays/ELISA kits following manufacturer instructions (15–17).

### Vascular function assessments

Brachial artery FMD was measured using a vascular probe on the Aplio i900 ultrasound system (Canon Ultrasound Systems, Melville, NY, USA), with a blood pressure cuff placed on the forearm and inflated to 220 mmHg for 5 min. Arterial diameter was recorded 1 min before cuff inflation (baseline) and 5 min after deflation (reactive hyperemia), and images were analyzed using automated edge-detection software. FMD (%) was calculated by subtracting the baseline diameter from the peak hyperemia diameter, dividing by the baseline value, and multiplying by 100 ^(^12, 13, 16–18). Microvascular reactivity was assessed in the biceps using Superb Microvascular Imaging (SMI), quantifying microvascular density and arteriolar diameters at baseline and after ischemia–reperfusion with analogous 5 min cuff occlusion, reporting percent changes from baseline. All vascular studies were acquired and analyzed by an experienced evaluator trained in vascular ultrasound, who was blinded to participant group assignment.

### Echocardiography

Myocardial function was assessed at rest using two-dimensional echocardiography on the Aplio i900 system (Canon Ultrasound Systems, Melville, NY, USA). LV volumes and EF were measured using Simpson's biplane method (19). Mitral inflow, tissue Doppler indices, and timing measures were recorded. Speckle-tracking analysis was performed using the Aplio i900 myocardial speckle-tracking and strain measurement package to calculate global longitudinal strain from the apical 4-, 3-, and 2-chamber views (20). Echocardiographic image acquisition and data analysis were performed by a certified echocardiographer who was blinded to participant clinical status and PASC diagnosis at the time of image acquisition and analysis.

### Statistical analysis

Given the exploratory pilot design and limited sample size, formal *a priori* power calculation was not performed; therefore, emphasis was placed on the magnitude and direction of between-group differences, with findings interpreted as hypothesis-generating rather than definitive. Continuous variables are presented as mean ± SD and categorical variables as *n* (%). Normality of continuous variables was assessed using Shapiro–Wilk testing together with visual inspection of histograms/Q–Q plots. Because several variables deviated from normality and group sizes were modest and unequal, group comparisons for most continuous outcomes were performed using Kruskal–Wallis tests; categorical outcomes were compared using chi-square tests, applying the Haldane–Anscombe correction for sparse cells when needed. *post-hoc* pairwise Wilcoxon rank-sum tests were used following significant omnibus tests. Echocardiographic outcomes were compared using Welch's ANOVASpearman correlation assessed relationships among pathway markers, endothelial function, inflammation/coagulation indices, and cardiac outcomes; *p*-values were unadjusted given exploratory analyses. Multivariable linear regression for brachial FMD% included the following prespecified covariates: age, BMI, NOx, ADMA, LDL, total cholesterol, eGFR, EF, TGF-β, microvascular FMD%, DDAH, group status, and race. These variables were selected based on biological relevance to endothelial function. Given the limited sample size relative to the number of predictors, this analysis was considered exploratory and hypothesis-generating, and the resulting estimates were interpreted cautiously. Exploratory machine-learning used a multilayer perceptron with 5-fold cross-validation, comparing performance against linear regression, and permutation importance ranked predictors (Supplementary Appendix). Analyses were conducted using SPSS v26.

## Results

### Patients characteristics

Across 49 participants (Controls *n* = 10, PASC− *n* = 20, PASC + *n* = 19), baseline age, BMI, blood pressure, heart rate, and central adiposity did not differ significantly (all *p* > 0.20). Lipids were higher in PASC+ (total cholesterol 193.2 ± 40.4 mg/dL, LDL 113.6 ± 40.4 mg/dL) than Controls (total cholesterol 146.5 ± 19.0, LDL 79.7 ± 12.7). Kidney function was modestly lower in PASC+ (eGFR 93.5 ± 15.4 vs. 110.8 ± 22.7 mL/min/1.73 m² in Controls; *p* = 0.048). Inflammation and coagulation indices were highest in PASC+: D-dimer was >3-fold higher than Controls (0.7 ± 0.8 vs. 0.2 ± 0.05 μg/mL FEU; *p* = 0.001) and hs-CRP was ∼3-fold higher (3.4 ± 1.6 vs. 1.2 ± 0.7 mg/L; *p* = 0.002), with PASC− generally intermediate. Full baseline characteristics are presented in Table 1.

**Table 1:** Patient characteristics.

Characteristic		Overall	Control	PASC-	PASC+	*p* value
Total participants, *n*		49	10	20	19	—
Male, *n* (%)		16 (32.7%)	5 (50.0%)	7 (35.0%)	4 (21.1%)	0.275
Age, years		37.8 ± 9.1	35.9 ± 11.9	36.1 ± 8.7	40.6 ± 7.5	0.2369
Race	Non-Hispanic White	20 (40.8%)	2 (20.0%)	8 (40.0%)	10 (52.6%)	0.266
	African American	15 (30.6%)	6 (60.0%)	6 (30.0%)	3 (15.8%)	
	Hispanic	9 (18.4%)	0 (0.0%)	4 (20.0%)	5 (26.3%)	
	Asian	5 (10.2%)	2 (20.0%)	2 (10.0%)	1 (5.3%)	
Anthropometric outcomes	BMI, kg/m²	31.5 ± 8.6	32.0 ± 10.1	32.4 ± 8.7	30.2 ± 7.9	0.7187
	Waist circum, cm	96.5 ± 19.4	101.5 ± 20.1	96.3 ± 12.4	94.0 ± 24.8	0.7890
	Hip circum, cm	103.9 ± 18.1	110.9 ± 20.8	103.5 ± 13.7	100.6 ± 20.6	0.5210
	Waist/Hip Ratio	0.9 ± 0.1	0.9 ± 0.1	0.9 ± 0.1	0.9 ± 0.1	0.9755
Glycemic parameters	Fasting plasma glucose, mg/dL	82.8 ± 10.8	87.3 ± 10.0	82.8 ± 10.7	80.3 ± 11.1	0.2083
	HbA1c, %	5.4 ± 0.4	5.5 ± 0.3	5.4 ± 0.4	5.3 ± 0.4	0.5466
Lipid profile	Cholesterol, mg/dL	175.10 ± 37.70	146.50 ± 19.01	172.25 ± 33.23	193.16 ± 40.44	0.002
	Triglycerides, mg/dL	99.63 ± 47.84	101.40 ± 44.19	88.45 ± 26.74	110.47 ± 64.08	0.653
	HDL, mg/dL	54.65 ± 16.83	46.50 ± 13.59	56.00 ± 15.15	57.53 ± 19.31	0.142
	LDL, mg/dL	104.04 ± 34.00	79.70 ± 12.67	98.50 ± 25.16	113.63 ± 40.43	0.023
Liver parameters	Total Bilirubin, mg/dL	0.63 ± 0.40	0.74 ± 0.40	0.64 ± 0.50	0.66 ± 0.27	0.294
	Alkaline Phosphatase, U/L	67.29 ± 22.43	69.90 ± 25.39	62.05 ± 15.53	67.16 ± 20.62	0.672
	AST, U/L	20.31 ± 5.19	21.40 ± 5.68	16.15 ± 3.59	19.37 ± 5.45	0.097
	ALT, U/L	20.12 ± 9.59	19.70 ± 6.52	17.45 ± 5.95	20.11 ± 10.85	0.423
Cardiac parameters	Systolic BP, mmHg	117.7 ± 15.1	120.4 ± 19.1	115.2 ± 8.4	118.8 ± 18.5	0.7429
	Diastolic BP, mmHg	77.7 ± 7.1	75.6 ± 6.8	76.8 ± 6.2	79.8 ± 8.0	0.4507
	Heart rate, bpm	74.5 ± 10.3	76.6 ± 10.1	73.0 ± 10.4	74.9 ± 10.5	0.5048
Blood parameters	Neutrophils, ×10³/µL	4.02 ± 1.45	4.14 ± 1.15	3.88 ± 1.43	4.18 ± 1.22	0.167
	Lymphocytes, ×10³/µL	2.01 ± 0.69	2.13 ± 0.60	1.95 ± 0.47	1.83 ± 0.61	0.078
	N/L ratio	2.15 ± 1.01	2.39 ± 1.23	2.01 ± 0.79	2.57 ± 1.54	0.059
Renal parameters	Bun, mg/dL	13.2 ± 2.9	14.5 ± 2.7	13.0 ± 2.2	12.7 ± 3.5	0.2398
	Creatinine, mg/dL	0.8 ± 0.2	0.8 ± 0.2	0.8 ± 0.2	0.9 ± 0.2	0.3925
	Cystatin C, mg/L	0.8 ± 0.1	0.9 ± 0.2	0.8 ± 0.1	0.8 ± 0.1	0.1721
	eGFR, mL/min/1.73 m²	101.9 ± 18.9	110.8 ± 22.7	105.5 ± 17.4	93.5 ± 15.4	0.0481
	Vitamin D, ng/mL	28.5 ± 14.7	27.4 ± 20.6	25.6 ± 9.0	32.1 ± 16.1	0.4408
Inflammatory parameters	ACTH, pg/mL	30.02 ± 17.68	18.40 ± 10.41	35.67 ± 18.55	17.67 ± 8.05	<0.001
	Cortisol, µg/dL	11.46 ± 5.98	7.62 ± 2.93	13.89 ± 5.12	7.82 ± 2.08	<0.001
	D-dimer, μg/mL FEU	0.5 ± 0.5	0.2 ± 0.05	0.3 ± 0.2	0.7 ± 0.8	0.0010
	CRP, high sensitivity, mg/L	2.5 ± 1.7	1.2 ± 0.7	2.2 ± 1.8	3.4 ± 1.6	0.0024

PASC, post-acute sequelae of SARS-CoV-2 infection (PASC+ with PASC symptoms; PASC− without PASC); BMI, body mass index; circum, circumference; BP, blood pressure; bpm, beats per minute; HbA1c, glycated hemoglobin; HDL, high-density lipoprotein; LDL, low-density lipoprotein; AST, aspartate aminotransferase; ALT, alanine aminotransferase; hs-Troponin-1, high-sensitivity troponin I; NT-proBNP, N-terminal pro–B-type natriuretic peptide; Neutrophils and lymphocytes reported as ×10³/µL; N/L ratio, neutrophil-to-lymphocyte ratio; BUN, blood urea nitrogen; Cystatin C, cystatin C; eGFR, estimated glomerular filtration rate; ACTH, adrenocorticotropic hormone; D-dimer reported in µg/mL FEU (fibrinogen equivalent units); CRP, C-reactive protein. The *p*-values compare groups (Control vs. PASC− vs. PASC+) using Kruskal–Wallis for continuous variables and chi-square with Haldane–Anscombe correction for categorical variables.

### Vascular biomarker and endothelial function profiles across groups

**Figure 1:**
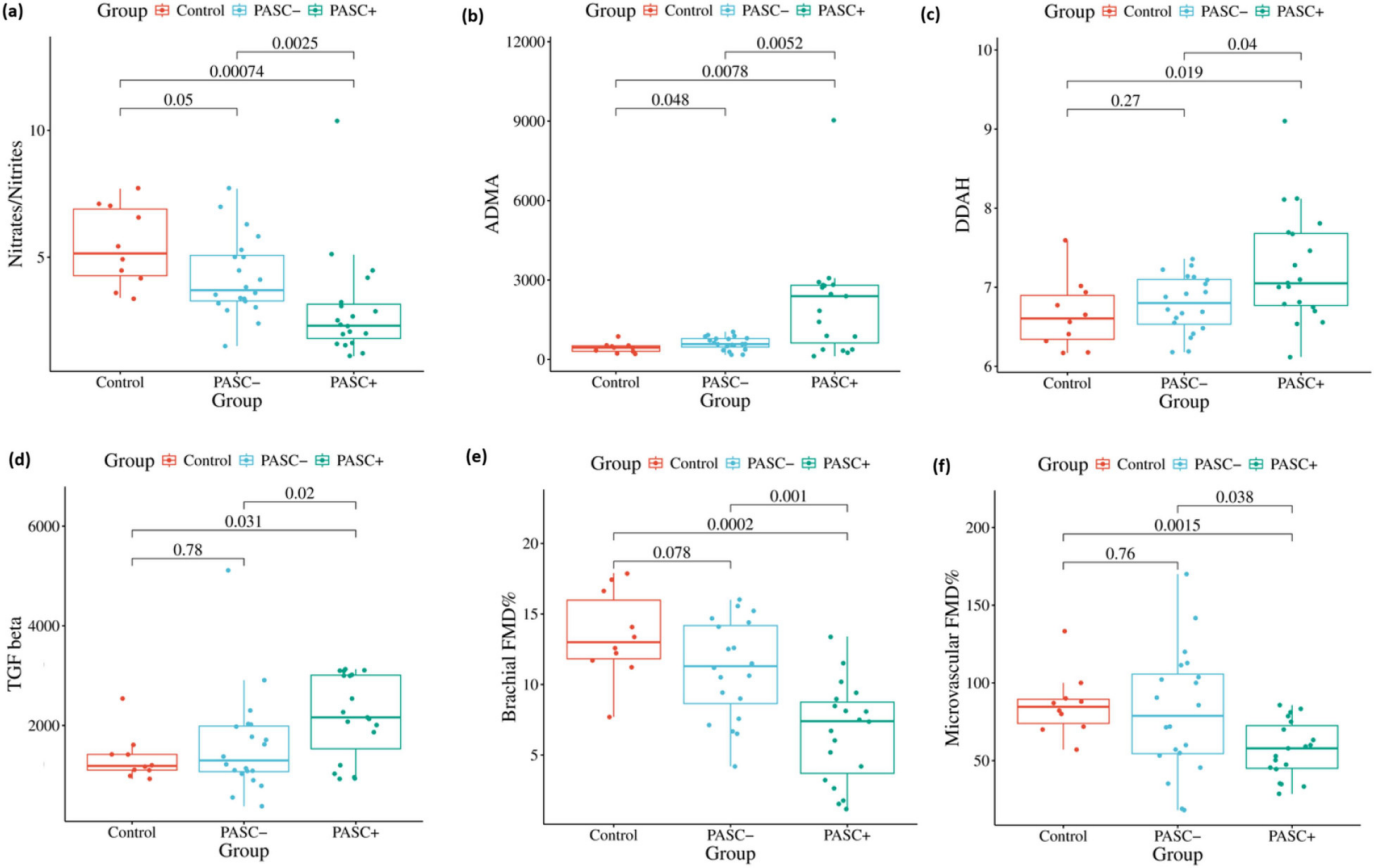
Dysregulation of the ADMA–DDAH–NOx pathway and endothelial dysfunction in PASC. Box-and-dot plots comparing Control, PASC–, and PASC+ groups. Panels show **(a)** Nitrates/Nitrites (NOx, μmol/L), **(b)** ADMA (nmol/L), **(c)** DDAH (units), **(d)** TGF-β (pg/mL), **(e)** Brachial FMD (%) and **(f)** Microvascular FMD (%). Boxes indicate median and IQR, whiskers indicate the range; dots are individual participants; and brackets display pairwise *p*-values using the Wilcoxon test.

PASC+ demonstrated marked disruption of the NO pathway and impaired endothelial function (Figure 1). Compared with Controls, PASC+ had lower NOx (3.0 vs. 5.4 μmol/L; *p* = 0.0007) and substantially higher ADMA (∼4.8-fold; 2,103 vs. 439 nmol/L; *p* = 0.0095). DDAH was modestly but significantly higher in PASC+ than in controls (approximately 7% increase; *p* = 0.0193), suggesting insufficient counter-regulation in the setting of lower NOx and higher ADMA. Functionally, endothelial reactivity was significantly impaired in PASC+: brachial FMD was reduced by ∼51% (6.6% vs. 13.5%; *p* = 0.0002) and microvascular FMD by ∼33% (57.2% vs. 86.0%; *p* = 0.0015). PASC− largely resembled Controls for brachial and microvascular FMD, while showing intermediate biomarker profiles. TGF-β was elevated in PASC+ compared with Controls (*p* = 0.031). *post-hoc* Pairwise testing showed consistently worse vascular biomarker and functional measures in PASC+ vs. PASC−.

### Echocardiographic profiles across groups

PASC+ exhibited worse myocardial mechanics and higher cardiac stress (Table 2). Averaged GLPS was impaired in PASC+ (−16.56 ± 3.17%) compared with Controls (−19.24 ± 0.93%) and PASC− (−18.49 ± 1.25%; *p* = 0.006). Electromechanical systole was prolonged in PASC + (403 ± 56 ms) compared with Controls (356 ± 30 ms; *p* = 0.002). LV end-diastolic volume was lower in PASC+ (76 ± 23 mL) than Controls (103 ± 29 mL; *p* = 0.029). EF differed across groups (*p* = 0.014), with substantially lower values in PASC+ (38.3 ± 16.57%) compared with Controls (53.4 ± 9.0%) and PASC− (49.5 ± 9.49%). The wide dispersion of EF values in PASC+ suggests heterogeneity, with a subset showing markedly reduced systolic function. Cardiac biomarkers were higher in PASC+ than comparison groups (hs-troponin-I and NT-proBNP).

**Table 2:** Echocardiographic profiles across groups.

Variable	Group	*N*	Mean	SD	SE	*P*
GLPS (A3C, %)	Control	10	−18.64	1.868	0.5907	**0**.**038**
	PASC+	19	−15.99	3.831	0.8788	
	PASC-	20	−18.70	2.732	0.6110	
GLPS (A4C, %)	Control	10	−18.93	1.543	0.4879	0.146
	PASC+	19	−17.54	3.237	0.7427	
	PASC-	20	−17.75	1.786	0.3994	
GLPS (A2C, %)	Control	10	−20.13	1.366	0.4321	**<**.**001**
	PASC+	19	−16.19	2.944	0.6753	
	PASC-	20	−19.05	1.869	0.4179	
GLPS (AVG, %)	Control	10	−19.24	0.925	0.2926	**0**.**006**
	PASC+	19	−16.56	3.171	0.7276	
	PASC-	20	−18.49	1.254	0.2803	
ES AVG (milliseconds)	Control	10	355.60	29.512	9.3324	**0**.**002**
	PASC+	19	403.26	55.828	12.8078	
	PASC-	20	344.35	28.529	6.3794	
EDV (mL)	Control	10	103.40	28.815	9.1120	**0**.**029**
	PASC+	19	76.32	23.226	5.3284	
	PASC-	20	93.20	24.421	5.4606	
ESV (mL)	Control	10	46.05	8.103	2.5624	0.480
	PASC+	19	52.64	20.589	4.7235	
	PASC-	20	46.88	9.611	2.1492	
EF (%)	Control	10	53.40	8.996	2.8449	**0**.**014**
	PASC+	19	38.3	16.54	3.8000	
	PASC-	20	47.95	9.49	2.1200	
LVLD DIFF (%)	Control	10	3.01	1.776	0.5618	**<**.**001**
	PASC+	19	9.97	4.367	1.0019	
	PASC-	20	4.76	3.559	0.7958	
LVLS DIFF (%)	Control	10	5.77	5.867	1.8553	0.054
	PASC+	19	10.09	4.600	1.0552	
	PASC-	20	6.50	5.474	1.2241	
S’ (cm/s)	Control	10	9.76	1.694	0.5357	0.123
	PASC+	19	8.52	1.828	0.4194	
	PASC-	20	9.63	1.948	0.4357	
IVCT (milliseconds)	Control	10	53.80	4.467	1.4126	0.069
	PASC+	19	59.74	9.700	2.2254	
	PASC-	20	53.35	8.506	1.9019	
IVRT (milliseconds)	Control	10	62.20	6.125	1.9368	0.145
	PASC+	19	74.26	24.454	5.6101	
	PASC-	20	62.60	11.691	2.6141	
Ea/Aa ratio	Control	10	1.53	0.254	0.0803	0.100
	PASC+	19	1.23	0.545	0.1250	
	PASC-	20	1.53	0.225	0.0504	
High Sensitivity Troponin-1 (ng/L)	Control	10	2.60	1.075	0.3399	**0**.**005**
	PASC+	19	5.26	2.864	0.6571	
	PASC-	20	3.25	1.970	0.4405	
proBrain Natriuretic Peptide (pg/mL)	Control	10	20.90	18.021	5.6988	**0**.**019**
	PASC+	19	57.63	48.284	11.0771	
	PASC-	20	34.80	17.310	3.8707	

GLPS, global longitudinal peak strain; A2C/A3C/A4C, apical 2/3/4 chamber view; AVG, average; ES, end systolic; EDV, end diastolic volume; ESV, end systolic volume; EF, ejection fraction; LVLD DIFF, left ventricular longitudinal diastolic strain difference; LVLS DIFF, left ventricular longitudinal systolic strain difference; S’, systolic mitral annular tissue Doppler velocity; IVCT, isovolumic contraction time; IVRT, isovolumic relaxation time; Ea/Aa, early to late diastolic annular velocity ratio; PASC, post acute sequelae of SARS CoV 2 infection; SD, standard deviation; SE, standard error; N, sample size. *P*-values were calculated using Welch's one-way ANOVA for comparisons across Control, PASC−, and PASC+ groups.

Bold is statistical significance (*p* < 0.001).

### Correlation analyses

**Figure 2:**
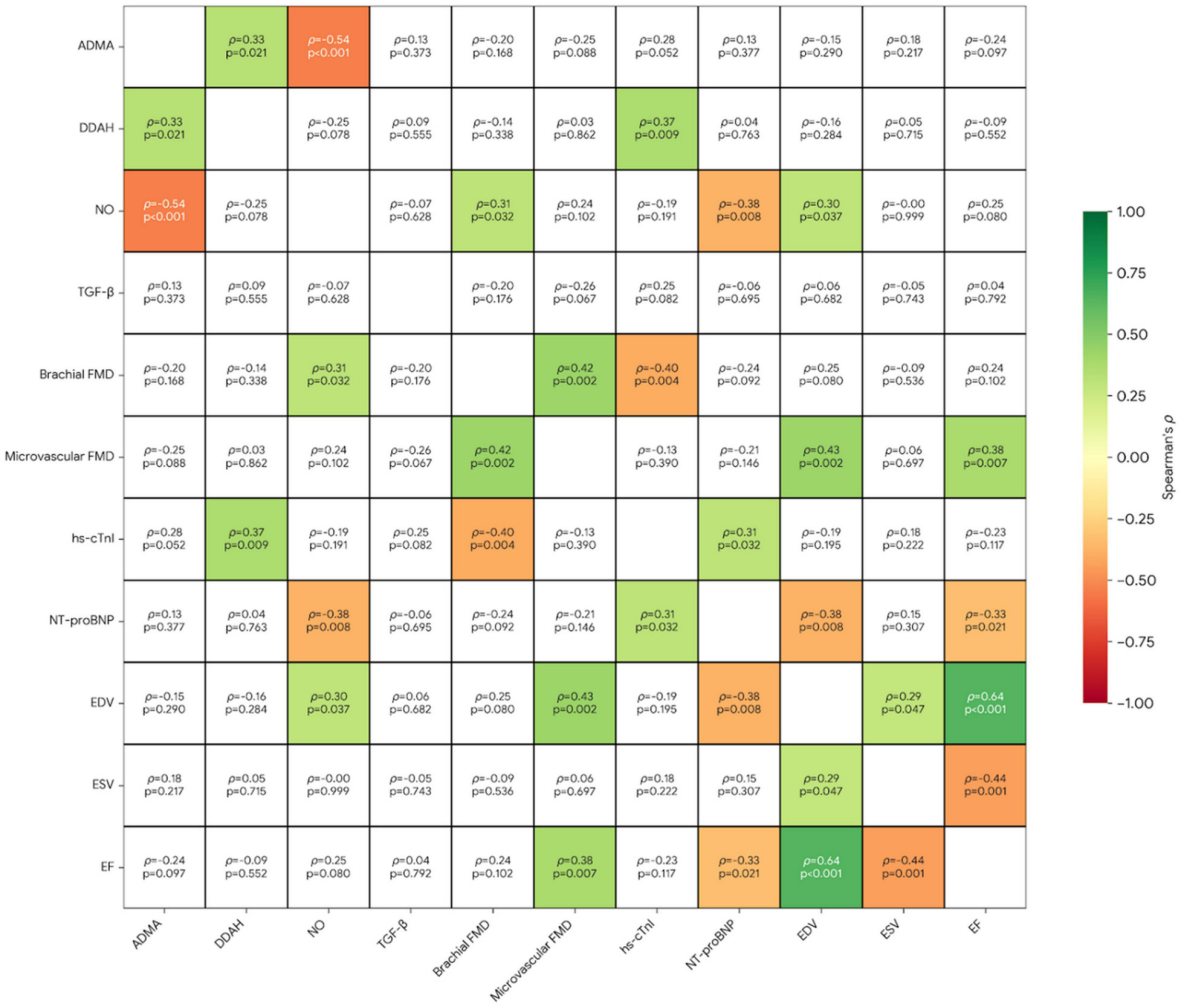
Correlation matrix linking vascular, inflammatory, and cardiac measures. Spearman correlation heatmaps of ADMA–DDAH–NO pathway markers, TGF-β, endothelial function, and echocardiographic/heart outcomes. Each cell shows Spearman's *ρ* and *p*; green marks significant positive correlations (*p* < 0.05), red marks significant negative correlations (*p* < 0.05), and uncolored cells are non-significant (*p* ≥ 0.05). Diagonal cells are omitted; *p*-values are two-sided and unadjusted for multiple comparisons.

In exploratory analyses, EF tracked most strongly with chamber size and biomarker load, showing a clear positive association with EDV (*ρ* = 0.64, *p* < 0.001) and an inverse relationship with ESV (*ρ* = −0.44, *p* = 0.001) and NT-proBNP (*ρ* = −0.33, *p* = 0.021). Endothelial function measures were concordant, with brachial and microvascular FMD moving in the same direction (*ρ* = 0.42, *p* = 0.002), and microvascular FMD also aligning with more favorable LV indices (positive correlations with EDV and EF). Within the ADMA–NO axis, higher ADMA related to lower NO (*ρ* = −0.54, *p* < 0.001), and NO in turn tended to associate with better vascular performance and lower NT-proBNP. Finally, vascular dysfunction clustered with myocardial injury signaling: brachial FMD correlated inversely with hs-cTnI (*ρ* = −0.40, *p* = 0.004), and hs-cTnI correlated positively with NT-proBNP, consistent with a link between endothelial impairment and cardiac stress/injury (Figure 2). In subgroup analyses, the strongest within-group findings were DDAH–brachial FMD in controls, ADMA–DDAH and brachial–microvascular FMD in PASC−, and a TGF-β–brachial FMD positive correlation in PASC+ (Supplementary Appendix, Supplementary Figure S1).

### Multivariable regression: prediction of brachial FMD%

Brachial FMD was primarily associated with group status in the adjusted model, with little independent contribution from the demographic and biomarker covariates retained in the final analysis. Compared with controls, PASC+ had significantly lower brachial FMD (*β* = −6.92, *p* < 0.001), whereas PASC− was not significantly different from controls (*β* = −2.33, *p* = 0.097). Race effects were not statistically significant overall, although Asian participants showed a borderline higher brachial FMD compared with Black participants (*β* = 4.05, *p* = 0.056); White vs. Black and Hispanic vs. Black comparisons were non-significant. Age, BMI, and DDAH were also not independently associated with brachial FMD (all *p* > 0.05). Overall model fit was moderate (R² = 0.504; adjusted R² = 0.405; F = 5.09, *p* < 0.001), suggesting that while the model explained a meaningful proportion of variance in brachial FMD, the principal signal was driven by symptomatic PASC status rather than the other included covariates. Given the modest sample size, these estimates should still be interpreted as exploratory (Table 3).

**Table 3:** Multivariable regression analysis for brachial FMD%.

Model Coefficients—Brachial FMD%						
Predictor	*β*	SE	95% Confidence Interval	*t*	*p*	
Intercept^a^	7.66769	7.7536	[−8.003, 23.338]	0.9889	0.329	
Age	−0.00760	0.0577	[−0.124, 0.109]	−0.1317	0.896	
BMI	0.00786	0.0687	[−0.131, 0.147]	0.1144	0.910	
Race:						
Asian—Black	4.05366	2.0579	[−0.105, 8.213]	1.9698	0.056	
Hispanic—Black	1.09762	1.7197	[−2.378, 4.573]	0.6383	0.527	
White—Black	−0.13665	1.5025	[−3.173, 2.900]	−0.0909	0.928	
Group:						
PASC+—Control	−6.91559	1.5125	[−9.972, −3.859]	−4.5723	<.001	
PASC-—Control	−2.32843	1.3696	[−5.097, 0.440]	−1.7000	0.097	
DDAH	0.75819	1.0579	[−1.380, 2.896]	0.7167	0.478	

β, regression coefficient; SE, standard error; CI, confidence interval; BMI, body mass index; PASC, post-acute sequelae of SARS-CoV-2 infection; FMD%, flow-mediated dilation; DDAH, dimethylarginine dimethylaminohydrolase.

R = 0.71, R^2^ = 0.504, Adjusted R^2^ = 0.405.

F = 5.09, *P* = <.001.

a Represents reference level.

## Discussion

Our findings demonstrate that individuals with persistent post-acute sequelae of COVID-19 (PASC+) exhibit a distinct pattern of vascular and cardiac dysfunction compared with both PASC− and never-infected Controls. The key finding is the dysregulation of the ADMA–DDAH–NO*x* axis in PASC+, characterized by markedly elevated ADMA levels, reduced NO_x_ metabolites, and impaired FMD. This indicates pronounced endothelial dysfunction in the PASC+ group, whereas PASC− individuals showed intermediate alterations between Controls and PASC+. However, because PASC+ was defined by cardiovascular-related symptoms, these findings should be interpreted as characterizing a cardiovascular-symptom long-COVID phenotype rather than long COVID broadly.

Notably, this is the first study to assess DDAH in the context of long COVID; although DDAH levels were modestly increased in PASC+, it was insufficient to offset the substantial accumulation of ADMA. In parallel, PASC+ individuals exhibited signs of persistent inflammation and coagulation activation, as well as significant cardiac impairment, including reduced ejection fraction, global longitudinal strain, and elevated troponin-I and pro-BNP levels.

Our results corroborate and extend emerging evidence that long COVID is associated with lasting endothelial dysfunction. For example, Ambrosino *et al*. (1) reported significantly reduced brachial artery FMD percentage in convalescent COVID-19 patients two months after infection (mean 3.2%) compared with matched controls (6.4%). Consistent with these observations, we found a 51% reduction in brachial FMD in PASC+ relative to controls, with PASC− showing intermediate values. Ambrosino's study noted that this effect was “sex-biased,” with male patients driving the FMD reduction. Although a trend toward worse FMD was observed in males in our cohort, the limited sample size and the higher proportion of females in the PASC+ group precluded definitive conclusions regarding sex differences. Nonetheless, the consistent FMD reduction across studies underscores that endothelial dysfunction is a significant feature of post-COVID conditions (2, 3). Importantly, our PASC+ group included individuals who initially had mild illness but developed long-term symptoms, indicating that significant endothelial impairment can persist for months after infection, even in non-hospitalized patients, thereby extending prior reports that primarily focused on severe or ICU-treated COVID-19 cases (1, 4).

Mechanistically, our data suggest that persistent inflammation and immune activation after COVID-19 perpetuate endothelial injury (4). PASC+ participants exhibited nearly threefold higher CRP levels than controls and the highest ADMA concentrations, exceeding control levels by more than fourfold. Since ADMA is a potent endogenous inhibitor of endothelial nitric oxide synthase (eNOS), its elevation reduces NO bioavailability and thus impairs endothelium-dependent vasodilation (5). Prior studies have shown that inflammation and oxidative stress can lead to ADMA accumulation by both increasing its generation via protein breakdown and by inhibiting its clearance via DDAH (6, 7). Consistent with this paradigm, ADMA in our study correlated positively with DDAH and inversely with NOx, supporting an ADMA–DDAH–NO*x* axis that links dysregulated methylarginine metabolism to impaired nitric-oxide bioavailability and vascular dysfunction. While elevated ADMA has been associated with severity and mortality in acute COVID-19 (8), our data show that this perturbation persists in the long-term post-acute sequelae, with PASC+ individuals exhibiting ADMA concentrations exceeding those reported in healthy controls or acute illness, highlighting a sustained nitric oxide deficit state.

Importantly, we examined DDAH, the enzyme responsible for more than 80% of the metabolic degradation of ADMA (6), and to our knowledge, this is the first evaluation of DDAH in PASC. We observed a modest increase in plasma DDAH levels in PASC+ individuals, suggesting a partial compensatory response to ADMA elevation. Although this difference reached statistical significance, its absolute magnitude was small, especially relative to the marked increase in ADMA and reduction in NOx. This pattern suggests that any compensatory upregulation of circulating DDAH was biologically insufficient to restore pathway balance. One possibility is reduced effective DDAH activity and/or altered compartmentalization despite higher circulating DDAH levels; enzymatic activity was not measured in this study. Oxidative and nitrosative stress can post-translationally modify DDAH and impair its enzymatic activity, such that an inflammatory milieu can blunt DDAH's ability to clear ADMA, even if the enzyme is upregulated (9–11). Our findings are consistent with this scenario; PASC+ had both the highest oxidative-inflammatory markers and the highest ADMA, indicating that DDAH-mediated ADMA clearance was insufficient. This imbalance between ADMA production and clearance is a key mechanistic insight of our study. Similar phenomena have been described in other disease states; for instance, sepsis models show that knockdown of DDAH causes a surge in ADMA and endothelial dysfunction, whereas overexpression of DDAH or administration of exogenous DDAH enzyme lowers ADMA and improves outcomes (12–14).

Our findings also align with the pro-thrombotic state previously described in PASC. In our cohort, the average D-dimer levels were more than three times higher in PASC+ compared to controls, consistent with prior reports of persistently elevated D-dimer and fibrinogen levels in long COVID, which suggest ongoing microthrombosis or impaired fibrinolysis (15). Townsend et al. (2021) (16) described a state of persistent endotheliopathy in long COVID, characterized by prolonged elevation of von Willebrand factor (VWF) and D-dimer up to 4 months post-infection. Endothelial dysfunction and reduced NOx bioavailability provide an explanation for this coagulation abnormality, as NOx deficiency promotes platelet activation, adhesion molecule expression, and a pro-coagulant endothelial phenotype (17).

In parallel, TGF-β was also elevated in PASC+ participants. TGF-β is a cytokine associated with fibrosis and endothelial-to-mesenchymal transition; its increase in PASC+ could signal incipient vascular remodeling or cardiac fibrosis (18). Recent work has highlighted vascular fibrosis and glycocalyx damage in post-COVID syndrome as potential mechanisms of long-term vascular stiffness (19).

With respect to cardiac function, PASC+ participants had notable impairments. Global longitudinal strain was reduced by approximately 10%–14% compared to PASC− and controls, and left ventricular ejection fraction (LVEF) was lower on average. These findings are consistent with emerging evidence from advanced cardiac imaging in post-COVID populations. For example, Roca-Fernandez et al. (20) reported cardiac MRI abnormalities in one in five individuals with Long COVID at 6 months, persisting in more than half of them at 12 months. Similarly, a study using speckle-tracking echocardiography detected subtle reductions in myocardial strain in recovered COVID patients despite preserved LVEF (21). Our data extend these observations by indicating that myocardial involvement in PASC+ can be clinically meaningful in a subset of patients, as several individuals exhibited markedly reduced ejection fraction. These findings raise the possibility of antecedent myocarditis, stress cardiomyopathy during acute infection, or the development of tachycardia-mediated cardiomyopathy during the post-acute phase (22), although these mechanisms cannot be confirmed in the absence of pre-COVID cardiac imaging.

Although pre-COVID cardiac imaging was unavailable, the reduced EF observed in a subset of symptomatic PASC participants is biologically compatible with post-viral myocardial injury (23). In biopsy-based viral heart disease, persistence of viral genomes in the myocardium has been associated with progressive LV dysfunction. LVEF declined from 54.3% ± 16.1% to 51.4% ± 16.1% when viral genomes persisted whereas viral clearance was associated with improvement in LVEF from 50.2% ± 19.1% to 58.1% ± 15.9% (24). In a nationwide Japanese registry of biopsy-proven fulminant myocarditis, 22 of 153 patients (14%) still had LVEF < 50% at 1 year, and reduced LVEF at discharge was associated with a markedly higher adjusted risk of death or heart transplantation (HR 8.19, 95% CI 2.13–31.5) (25). Case-level reports also support progression from viral myocarditis to clinically important systolic dysfunction, including one patient whose EF declined from 55%–60% to 35%–40% over 5 months without clinically significant coronary artery disease and a COVID-19–related cardiomyopathy case presenting with LVEF 20%–25% (26, 27). These data support low EF in our cohort as a component of post-COVID cardiovascular involvement, while not excluding previously unrecognized pre-existing dysfunction.

Consistent with these findings, PASC+ participants showed higher levels of hs-troponin and proBNP, indicating ongoing myocardial injury and stress. Endothelial-derived NOx is a key regulator of coronary microvascular tone and myocardial oxygen delivery; sustained NOx deficiency could therefore promote microvascular ischemia or diastolic dysfunction, ultimately affecting global cardiac performance (28). In parallel, inflammatory mediators associated with ADMA elevation may directly depress myocardial contractility or induce fibrotic remodeling. Thus, PASC appears to involve a feed-forward loop of multisystem dysfunction centered on vascular pathology, an insight that aligns with the emerging concept of long COVID as a predominantly vascular syndrome (29).

Several limitations of our study should be acknowledged. First, the sample size was modest and feasibility-based, reflecting the number of eligible RECOVER participants available for this nested cross-sectional analysis at the UIC site; this small sample size along with single geographic recruitment area limit statistical power and the generalizability of findings to broader and more diverse populations. Given the modest sample size relative to the number of covariates, multivariable models should be interpreted as exploratory and may be prone to overfitting and coefficient instability. For instance, the large variability in EF within the PASC+ group suggests heterogeneity, with some individuals having normal cardiac function while others having markedly reduced EF; larger cohorts will be needed to identify predictors for these cardiac phenotypes. Because this was a small nested substudy with limited numbers of eligible participants, especially in the never-infected control group, formal individual matching was not feasible without substantial loss of sample size and statistical informativeness. Second, the cross-sectional design of this study captures a single time point in the post-acute phase, precluding inference about causality or temporal changes. Third, although our control group was matched on key demographic variables, and participants were generally young with few comorbidities, residual confounding from unmeasured factors or unrecognized preexisting conditions, cannot be excluded. Our study also did not include a pre-COVID baseline for the infected groups, so we rely on parallel controls rather than intra-subject comparisons. We also cannot fully exclude the possibility of prior asymptomatic SARS-CoV-2 infection among controls despite protocol-based classification and negative testing at the index date.

Another limitation is related to our biomarker measurements, as we assessed ADMA and DDAH levels in circulating blood. These may not perfectly reflect tissue-level conditions. ACTH and cortisol are diurnal and stress-sensitive; because sampling conditions and intercurrent exposures can influence these measures, group differences in these hormones should be interpreted cautiously. DDAH exists in two isoforms (DDAH1 predominantly in endothelium and liver, DDAH2 in endothelium and immune cells); our assay reflects overall level and cannot identify tissue-specific contributions (11). We also did not quantify symmetric dimethylarginine (SDMA) or monomethylarginine (MMA), which, along with ADMA, can influence NOx pathways (30, 31). Finally, our study focused primarily on cardiovascular biomarkers and did not evaluate other proposed mechanisms of PASC, such as autoimmunity, viral persistence, or detailed organ-specific imaging. Given the multifactorial nature of PASC, our vascular-centered approach captures only one dimension of the syndrome. Integration with neurocognitive, pulmonary, or other systemic assessments would provide a more comprehensive understanding, but it was beyond the scope of this study.

## Conclusion and future perspectives

Long COVID is characterized by a sustained disruption of the ADMA–DDAH–NO*x* axis and persistent endothelial dysfunction that distinguishes individuals with PASC from those who recover fully and from never-infected controls. PASC+ participants exhibited elevated ADMA and inflammatory and prothrombotic markers, reduced nitric oxide bioavailability, impaired vascular function, and evidence of heterogeneous cardiac involvement, including reduced systolic function in a subset. These findings identify defective DDAH-mediated ADMA clearance as a previously unrecognized mechanism in PASC, underscoring actionable biological targets for interventions aimed at restoring endothelial function and mitigating long-term cardiovascular risk.

Our findings open up several promising avenues for future research and intervention. A paramount question is whether targeting the ADMA–NOx pathway can ameliorate PASC symptoms or prevent long-term complications*.* Given that we identified a clear deficiency in NOx bioavailability in PASC+, therapies aimed at boosting NOx or reducing ADMA are warranted for exploration. One such approach is L-arginine supplementation. In fact, recent clinical trials have demonstrated that a 28-day course of oral L-arginine (1.6 g twice daily) combined with vitamin C significantly improves endothelial function and exercise capacity in patients with long COVID, compared to a placebo (8). This improvement was accompanied by an increase in the L-arginine/ADMA ratio, suggesting that restoring the substrate/inhibitor balance of NOx synthesis was beneficial. These findings align with our mechanistic data, suggesting that augmenting arginine availability may offset ADMA-mediated inhibition of NOx and potentially alleviate symptoms such as fatigue or vascular headaches (32). Similarly, exercise training is a non-pharmacologic strategy known to upregulate endothelial NOS and DDAH expression over time. In a recent randomized study, combined aerobic and resistance exercise training in patients with PASC effectively restored endothelial function and improved symptom scores (33). Together, these observations support targeted lifestyle and metabolic interventions as feasible, low-cost strategies to disrupt the cycle of endothelial dysfunction in long COVID.

Another promising therapeutic option is sulodexide, a glycosaminoglycan with anticoagulant and endothelial-protective properties. In the TUN-EndCOV study, Charfeddine et al. (34) showed that a 3-week course of sulodexide in long COVID patients significantly improved FMD and reduced symptoms like chest pain and palpitations. In parallel, sulodexide-treated patients had reductions in key biomarkers of endothelial injury and inflammation, including soluble thrombomodulin, vWF, IL-6, CRP, and D-dimer over 8 weeks of therapy (35). Notably, elevations in these same biomarkers were a defining feature of the PASC+ group in our cohort. Thus, these findings suggest that therapies aimed at preserving endothelial integrity and limiting microthrombosis directly target the pathophysiological features identified in this study. Future larger controlled trials of sulodexide or similar agents, such as rivaroxaban or antiplatelet therapy targeting microclots, are needed to determine whether endothelial restoration leads to a meaningful clinical benefit in long COVID.

From a mechanistic research standpoint, a compelling direction would be to test direct ADMA-lowering therapies. Although such approaches are not yet available clinically, experimental studies demonstrated that the administration of recombinant DDAH enzyme can reduce ADMA levels and protect organs in ischemia models (14, 36). It is conceivable that in the future, we could pharmacologically enhance DDAH activity or expression through gene therapy or small molecules that activate Nrf2, a known regulator of DDAH1 (37). An engineered ADMA-metabolizing enzyme (also called “M-DDAH”) has shown efficacy in reducing ADMA levels in preclinical studies (38). Should these strategies advance to human studies, PASC would represent a compelling target, given the prominent role ADMA seems to play. In the near term, more accessible interventions, including dietary approaches that reduce oxidative inhibition of DDAH (39) or folate and B-vitamin supplementation to lower homocysteine, which can inhibit DDAH (40), warrant exploration. Importantly, our results underscore the value of incorporating ADMA and DDAH measurements into future interventional studies of long COVID to determine whether clinical improvements parallel restoration of this pathway.

From a risk stratification standpoint, our results suggest that measuring markers of endothelial function in the convalescent phase of COVID may help identify those at risk for PASC or future cardiovascular issues. For instance, elevated ADMA or low NOx levels a few weeks post-infection could potentially predict the development of long COVID symptoms, a hypothesis that warrants testing in prospective cohorts. Likewise, genetic predispositions may play an important role, as polymorphisms in the DDAH2 gene or in enzymes regulating arginine (such as arginase-1 or NOS3) could predispose certain individuals to persistent disruption of NOx signaling following a viral hit (41–43). Investigating these genetic factors may clarify interindividual variability in PASC risk and ultimately support more personalized prevention and treatment strategies.

Clinically, health providers managing long COVID patients should be aware of the potential contribution of endothelial dysfunction and consider monitoring basic cardiovascular indices. For example, symptoms such as unexplained tachycardia, exertional dyspnea, or chest discomfort in PASC may warrant an evaluation of endothelial function or biomarkers like D-dimer, rather than being attributed solely to anxiety or deconditioning. In line with recommendations from the European Society of Cardiology, some post-COVID clinics have begun incorporating measures of flow-mediated dilation or arterial stiffness, an approach supported by our findings (29). Moreover, interventions with established cardioprotective and endothelial benefits may offer dual advantages in this population. Statins, for instance, have pleiotropic effects that include improving endothelial function and upregulating DDAH activity (44); while speculative, a trial of low-dose statins in long COVID could be considered for selected long COVID patients, particularly those with elevated ADMA or dyslipidemia. Overall, targeting endothelial health may represent a critical component of effective PASC management.

## Data Availability

The raw data supporting the conclusions of this article will be made available by the authors, without undue reservation.
