# Supplementary material for: Endothelial and cardiac dysfunction in long COVID With cardiovascular symptoms is associated with imbalance in the ADMA–DDAH–NOx pathway
Source: Front Cardiovasc Med. 2026 Apr 21;13:1802359. doi: 10.3389/fcvm.2026.1802359 (PMC13139065; doi:10.3389/fcvm.2026.1802359)
Supplement: Supplementary file 1 [file Datasheet1.docx]

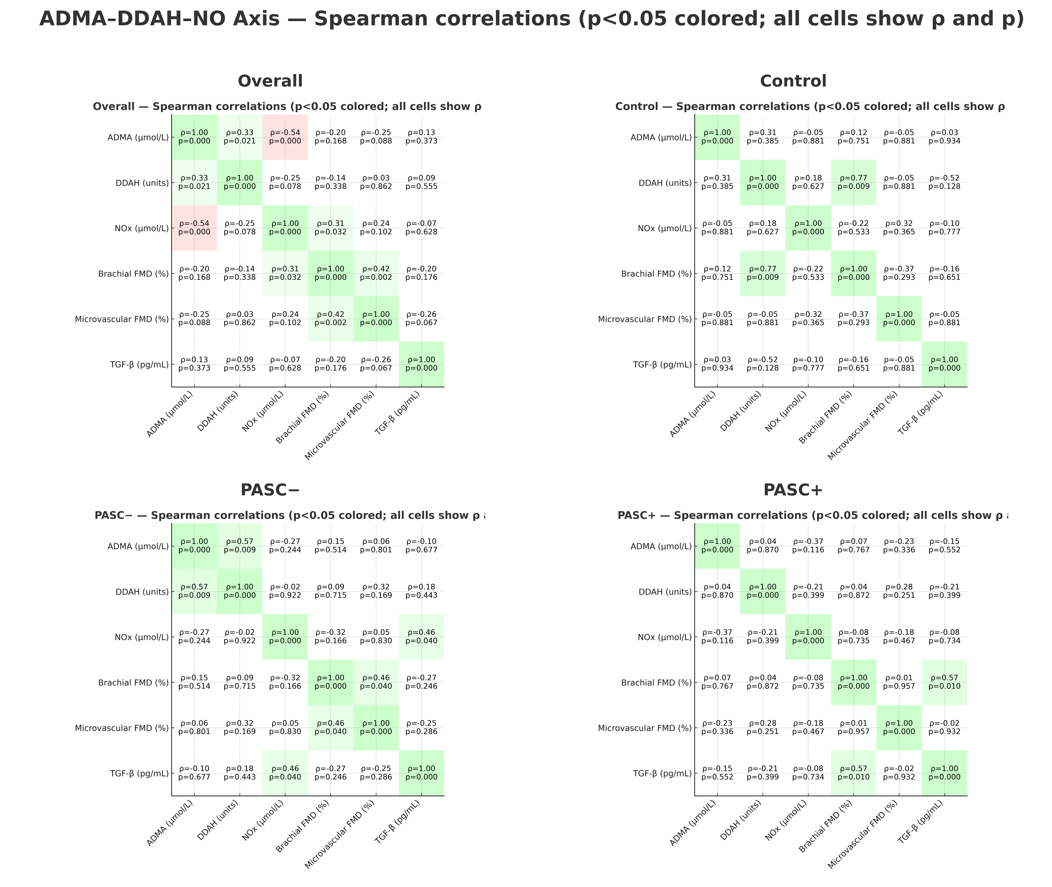


*Supplementary Figure 1. Spearman correlation heatmaps of ADMA–DDAH–NO pathway markers (ADMA, DDAH, NOx), endothelial function (brachial and microvascular FMD), and TGF-β shown for the overall cohort and stratified by Control, PASC−, and PASC+. Cells display Spearman’s ρ and two-sided p values; shading indicates statistically significant correlations (p<0.05). Diagonal cells are omitted.*

**Exploratory Neural Network: Prediction of Brachial FMD% and Microvascular FMD%**

When predicting brachial flow-mediated dilation (brachial FMD, %), a tuned multilayer perceptron (MLP) modestly outperformed linear regression, with slightly lower root-mean-square error (RMSE) (-1.1%) and mean absolute error (MAE) (-1.4%), and a higher coefficient of determination (R² = +34.6%). Similar improvements were observed for microvascular flow-mediated dilation (microvascular FMD, %), where the MLP achieved lower RMSE (-3.6%) and MAE (-5.8%) and higher R² (+17.3%) compared with linear regression models. Although modest, these differences may reflect limited non-linear signal in ADMA, DDAH, NOx, and related covariates; given the small sample, findings should be interpreted cautiously as hypothesis-generating **(Supplementary Figure 2)**. Given the small sample size, these machine-learning findings are hypothesis-generating and are not intended for clinical prediction.


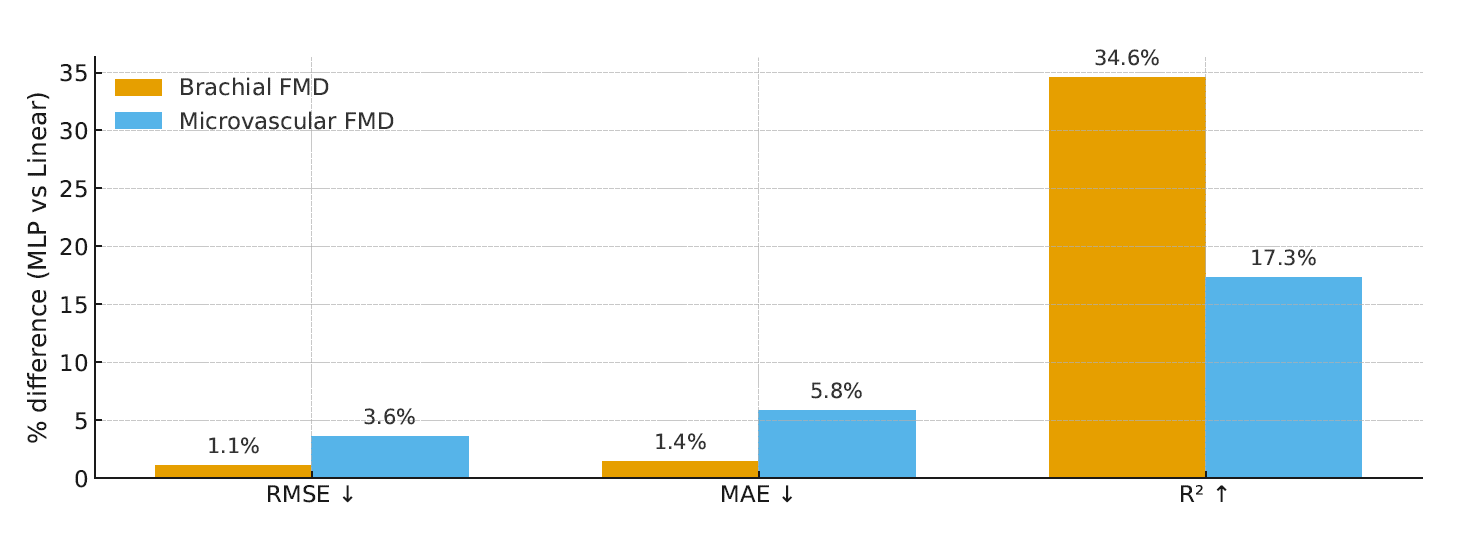


Supplementary Figure 2. Neural network versus linear model performance for endothelial function prediction. Bar chart showing percent improvement of an MLP over a linear model for predicting brachial and microvascular FMD (5-fold CV).

These improvements are supported by the out-of-fold observed-vs-predicted plots, where points cluster around the 45° line for both endpoints **(Supplementary Figure 3)**. Permutation importance highlights ADMA and NOx as top predictors, with DDAH and clinical covariates providing a secondary signal **(Supplementary Figure 4)**. Although effect sizes should be interpreted cautiously, given the small sample and limited feature set, the directionality is broadly consistent with biological plausibility, but should not be overinterpreted given the small sample and limited feature set.


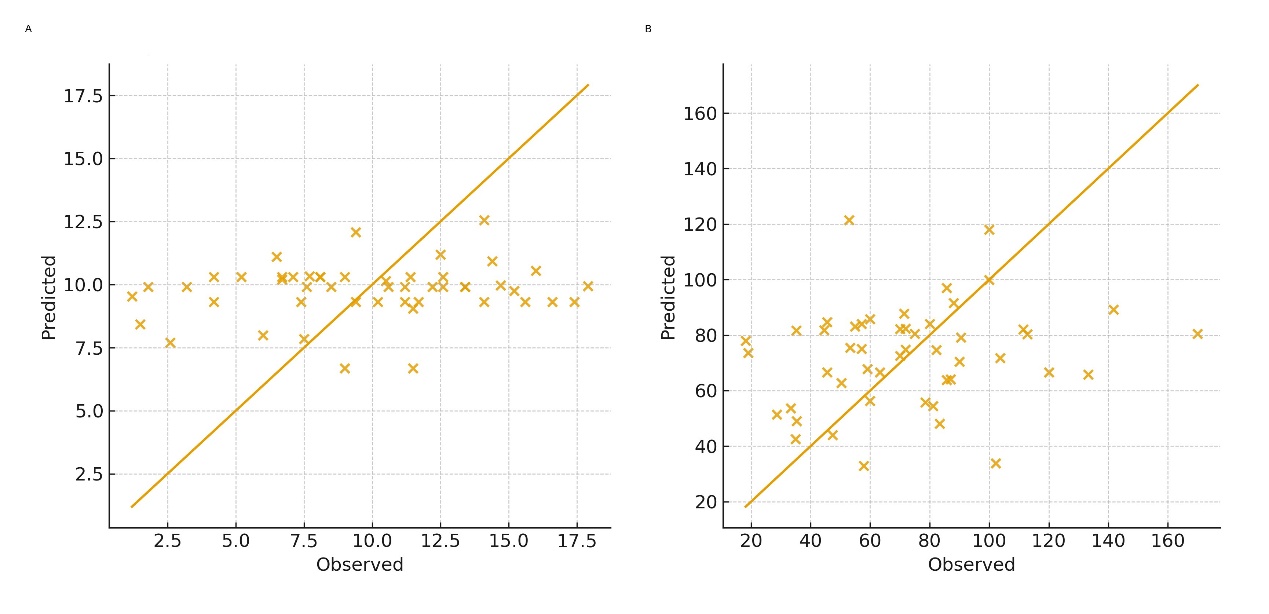


Supplementary Figure 3. Out-of-fold observed vs predicted scatterplots for brachial (A) and microvascular (B) FMD using an MLP with 5-fold CV; points cluster around the 45° line, with tighter alignment for brachial than microvascular.


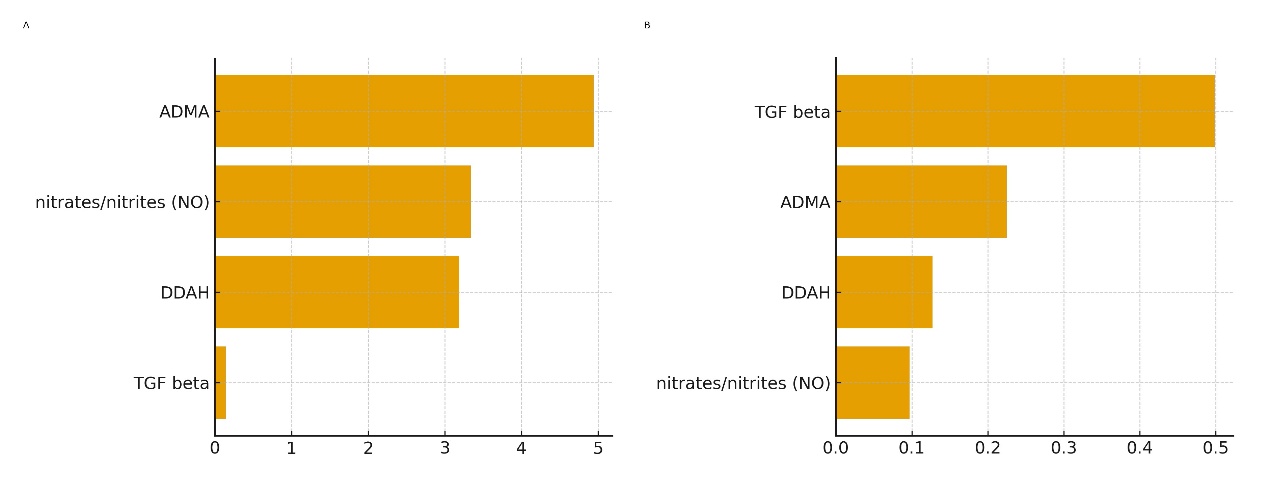


Supplementary Figure 4. Permutation-importance bar charts for the MLP; for brachial (A) and microvascular (B) FMD.
